# Uncover DNA damage and repair-related gene signature and risk score model for glioma

**DOI:** 10.1080/07853890.2023.2200033

**Published:** 2023-04-22

**Authors:** Yaqiu Wu, Ling Liu, Da Huang, Zhili Li, Ruxiang Xu, Meixiong Cheng, Longyi Chen, Qi Wang, Chao You

**Affiliations:** a Department of Neurosurgery, West China Hospital, Sichuan University, Chengdu, China; b Department of Neurosurgery Intensive Care Unit, Sichuan Provincial People’s Hospital, University of Electronic Science and Technology of China, Chengdu, China; c Department of Neurosurgery, Sichuan Provincial People’s Hospital, University of Electronic Science and Technology of China, Chengdu, China

**Keywords:** Glioma, DDR, risk score model, tumor, prognosis

## Abstract

**Background:**

Glioma is a common primary central nervous system tumor with complex pathogenesis. DNA damage and repair (DDR) is widely involved in regulating cell proliferation and tumorigenesis by correcting and repairing DNA damage mechanisms. Recent studies have reported the following properties in cancer cells in glioma, increased DNA damage and reduced DNA repair capacity. However, the relationship between glioma and DDR-related genes was unclear.

**Methods:**

DDR-related risk score model was built. The validity of this model was validated in detail through the Kaplan-Meier survival analysis, tumor mutational burden (TMB) analysis, immune cell infiltration, sensitivity to treatment regimens. Moreover, the model’s adaptability was validated in different glioma data cohorts and different glioma subgroups. To further investigate the molecular mechanism of one of DDR-related gene (NUDT1) in glioma, U251 cell was used for the knockdown experiment, followed by MTT, wound healing and transwell analysis.

**Results:**

Ten prognostic-related DDR-related signature genes were obtained, including EID3, MGMT, YWHAG, PMS1, SHPRH, HUS1, NUDT1, GADD45G, APEX1 and FAM175A. The RT-qPCR results suggested that the latter five genes were highly expressed in glioma patients. Interestingly, high TMB score had longer survival. In high-risk score groups, reduced immune cell infiltration in the tumor microenvironment lead to poorer patient outcomes. Sensitivity to treatment regimens analysis indicated that low-risk score groups were more sensitive to chemotherapeutics. Moreover, the risk score model had a good prediction effect on different glioma datasets and different glioma subgroups. *In vitro* mechanism study showed that knockdown of NUDT1 reduced tumorigenesis. Furthermore, knockdown of NUDT1 remarkably reduced the expression level of HIF-1α.

**Conclusion:**

DDR-related risk score model built-in this work has good predictive performance for glioma.

**Key messages**
Ten prognostic-related DDR-related signature genes were obtained, including EID3, MGMT, YWHAG, PMS1, SHPRH, HUS1, NUDT1, GADD45G, APEX1 and FAM175A.
In high-risk score groups, reduced immune cell infiltration in the tumor microenvironment leads to poorer patient outcomes.
The risk score model had a good prediction effect on different glioma datasets and different glioma subgroups.
Knockdown of NUDT1 reduced tumorigenesis of glioma and remarkably reduced the expression level of HIF-1α.

## 1. Introduction

Glioma is the most common adult brain tumor with the highest mortality rate [1]. Recently, therapeutic strategies of glioma rely on generating overwhelming DNA damage and inhibiting repair mechanisms [2]. The presence of glioma biomarkers may lead to specific susceptibility to DNA damage and repair inhibition. These biomarkers have been considered as eligibility criteria to design multiple clinical trials. Targeting DNA repair mechanisms are promising approach to treat gliomas [3,4]. Despite numerous studies on glioma, the key factors regulating glioma progression and malignant transformation remain unclear.

DNA damage and repair (DDR) is a crucial mechanism for correcting and repairing DNA damage, which can timely inhibit cell senescence and carcinogenesis [5]. DDR consists of 8 pathways. Interaction of DDR is able to accurately repair DNA damage and ensure genome integrity [6]. Recent studies have shown changes in the genomic stability of cells after they become cancerous, manifested by increased DNA damage and reduced DNA repair capacity [7]. The study of DDR-related genes was able to broaden treatment options for cancer patients. DDR alterations correlate with response to PD1/PD-L1 inhibitor therapy and are positively related to higher tumor mutational burdens [8].

Furthermore, it is suggested that tumors with deleterious DDR mutations were more sensitive to platinum-based therapy [9]. The alkylating agent temozolomide (TMZ) induces DNA breakage and subsequent cell death in glioma cells and is one of the main regimens used in glioma patients [4]. Thus, DDR-related genes play a key role in tumor resistance to chemoradiation.

DDR alterations are related to clinical and molecular characteristics of glioma. Overexpression of midkine (MDK), a key transcriptional factor in DDR pathways can remodel the glioma immunosuppressive microenvironment by promoting M2 polarization of microglia [10]. There are few studies on the interaction between DDR genes and the glioma tumor microenvironment and prognostic prediction [11,12]. Here, we obtained gene expression profiles of glioma from public databases. We further identified prognostic signature genes using differentially expressed genes (DEGs) related to DDR and validated the reliability of the model. We performed functional analysis to study its underlying mechanisms. In addition, we investigated the association between prognostic gene and immune infiltration type, explored the differences in clinical characteristics and tumor immune cell infiltration between different risk score groups in glioma, and further evaluated the risk score for the benefit of immunotherapy. In a short, the above data suggest that the risk score model can effectively predict the glioma prognosis.

## 2. Materials and methods

### 2.1. Data collection

Gene expression data were downloaded from The Cancer Genome Atlas (TCGA) database (involving RNA-seq expression profiling). TCGA database was used as the training set after log_2_(*x* + 1) processing. Three datasets were downloaded from the Chinese Glioma Genome Atlas (CGGA) database as validation sets [13]. Totally, 276 DDR-related genes were identified from the report of Knijnenburg et al. [14]. Among them, 265 DDR-related genes were identified in the TCGA cohort. All data were filtered to remove samples with missing clinical information.

### 2.2. Screening of DDR-related prognostic genes and construction of risk score model

In the TCGA cohort, DEGs were identified by the limma. |log_2_ fold change (FC)| > 2 and false discovery rate (FDR) < 0.05 was the screening condition of DEGs [15]. DDR-related genes with prognostic effects in glioma were identified through univariate Cox regression analysis. Further, the least absolute shrinkage and selection operator (LASSO) regression algorithm was used to construct a prognostic model [16]. The risk score was calculated.

According to the median risk score, patients were divided into the high-risk score and low-risk score groups. Survival analysis was performed using the survminer (R package) to analyze overall survival (OS) in high- and low-risk score groups [17]. The survminer and the timeROC were utilized to perform time-dependent ROC curve analysis [18]. Univariate and multivariate Cox analyses were used to assess the independent prognostic value of the risk score. Finally, the risk score was calculated through the same formula in the validation cohort.

### 2.3. Immune checkpoint analysis

Expression profiles of immune checkpoint-blockaded genes and associated clinical information were downloaded from public databases. Finally, an immunotherapy cohort (IMvigor210) was included: advanced urothelial carcinoma treated with the anti-PD-L1 antibody atezolizumab. Full expression data are available from the URL of http://research-pub.gene.com/imvigor210corebiologies. Raw count data were converted to transcripts per kilobase million. The CIBERSORT algorithm was used to estimate the infiltrated immune cell component using gene expression signatures.

### 2.4. Validity verifications of risk score model

All independent prognostic parameters and associated clinical parameters were constructed with stepwise Cox regression models to construct prognostic nomogram to predict 1-, 2-, and 3-year OS for glioma patients. A nomogram calibration curve was drawn to compare predicted versus observed OS.

### 2.5. Tumor mutational burden (TMB) analysis

The maftools was utilized to calculate the TMB score. Tumor samples were divided into high- and low-TMB score groups based on the median value of TMB score [19]. Survival analysis of different TMB groupings, gene mutation analysis, and correlation analysis were performed.

### 2.6. Immune infiltration analysis

The ssGSEA algorithm was chosen to quantify the relative abundance of each cell infiltrate in the LAML immune microenvironment [20]. The gene set marking each tumor immune microenvironment (TIME) infiltrating immune cell type enriched in multiple human immune cell subtypes was identified from Charoentong’s research [21]. Enrichment score was utilized to represent the relative abundance of each TIME infiltrating cell in each sample. ESTIMATE was utilized to calculate the immune score, stromal score, tumor purity, and ESTIMATE score for each glioma patient.

### 2.7. Chemosensitivity analysis

Based on the Genomics Drug Sensitivity of Cancer Database (GDSC), the *calcPhenotype* algorithm from oncoPredict (R package) was used to evaluate drug IC50 values for each sample in the training set [22]. To assess the correlation between drug sensitivity and risk score, the Spearman correlation between RiskScore and drug IC50 was calculated. The difference in small-molecule drug IC50 between high and low-risk score groups was compared.

### 2.8. Subgroup analysis

To gain an in-depth understanding of the pathogenic mechanism of glioma, the patients were divided into different subgroups based on different clinic-pathological characteristics, including age (≥ 60 and < 60), gender, etc. Fisher’s exact test was utilized to compare the differences in different clinical indicators between high- and low-risk groups. *P* value < 0.05 was regarded as statistically significant.

### 2.9. Real-time quantitative polymerase chain reaction (RT-qPCR)

Firstly, RNA was extracted from tissues by using TRIzol™ Reagent (Thermo Fisher, 15596026, Massachusetts, USA). The RNA was then reversed into cDNA by using FastKing RT Kit (TIANGEN, KR116, Beijing, China). All gene primers used are displayed in Table S1. RT-qPCR was performed by using ABI 7500 with SuperReal PreMix Plus Kit (TIANGEN, FP205, Beijing, China). The 2^−ΔΔCt^ method was utilized to calculate the relative expression level of the gene. ACTB was used as internal reference. This study was approved by the Ethics Committee of West China Hospital. All patients have provided informed consent.

### 2.10. Cell culture

To further investigate the molecular mechanism of one of DDR-related gene in the risk score model, U251 cell was used for knockdown experiment, followed by MTT, wound healing and transwell analysis. U251 cell was cultured in medium containing 10% of fetal bovine serum and 5% of CO_2_ at 37 °C incubator.

### 2.11. MTT analysis

The cells were digested by trypsin, counted, and adjusted to concentration of 1x10^4^ cells/mL. 100 uL of cell suspension was added to each well of the 96-well plate and cultured for 1, 2 and 3 days. 10uL of CCK-8 solution was added to the 96-well cell culture plate and incubated for 4 h. The absorbance at 450 nm was measured with a microplate reader.

### 2.12. Cell scratches and Transwell assay

Before the experiment, the cells were starved in a serum-free medium for 12h. The cells were digested with trypsin and centrifuged at 1000 rpm for 5 min. After removing the supernatant, the cells were washed with PBS. After the supernatant was discarded, the cells were suspended in a serum-free medium containing 0.1% of BSA for cell count. The cell density was adjusted to 2x10^5^/mL in a serum-free medium containing 0.1% of BSA. 500 uL of complete medium was added into 24-well plate. 100 uL of cell suspension was added in Transwell chamber. The cells were transferred to a 24-well plate containing the complete culture medium and incubated for 24h in the cell incubator. After removing the upper chamber medium, the cells were wiped with the moistened cotton swab. The bottom of Transwell chamber was immersed in 10% of the methanol solution to fix the cells for 30 s and transferred to pure water. After washing off the methanol, the bottom of Transwell chamber was immersed in crystal violet dye for dyeing for 2 min, and cleaned with pure water until the background was clear. Microscope photography was performed.

### 2.13. Statistical analysis

All statistics were performed using R software. The Wilcox test was used to screen for infiltrating immune cells with statistically different. Kaplan-Meier curves were drawn. Log-rank was utilized to test for significant differences in OS between groups. Univariate and multivariate Cox proportional hazards regression analyses were also performed to understand the relationship between risk score and OS. ROC analysis was used to evaluate the sensitivity and specificity of the risk score. The area under the ROC curve (AUC) was an indicator of the accuracy of the prognosis. In the RT-qPCR, paired t-test was used for statistical analysis.

## 3. Results

### 3.1. Construction of DDR-related risk score model

**Figure 1.**
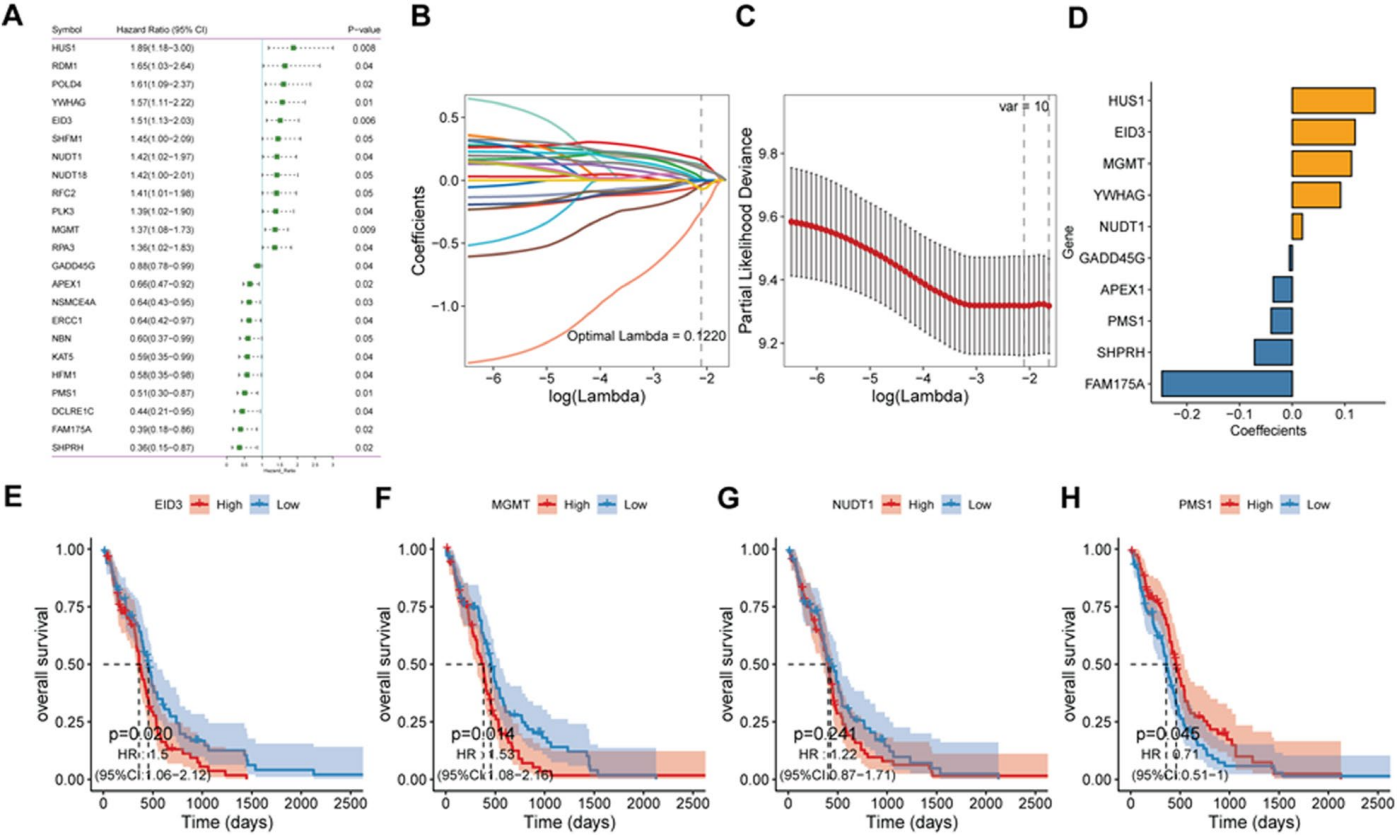
**Construction of risk score model**. A: prognosis-related gene identified by univariate Cox analysis. B: LASSO coefficient profiles of 23 candidate genes. C: screening of the best parameter (lambda) in the LASSO model. D: regression coefficients for 10 DDR-related signature genes. E–H, Kaplan-Meier survival analysis of EID3, MGMT, NUD1 and PMS1in the high- and low-risk score groups.

**Figure 2.**
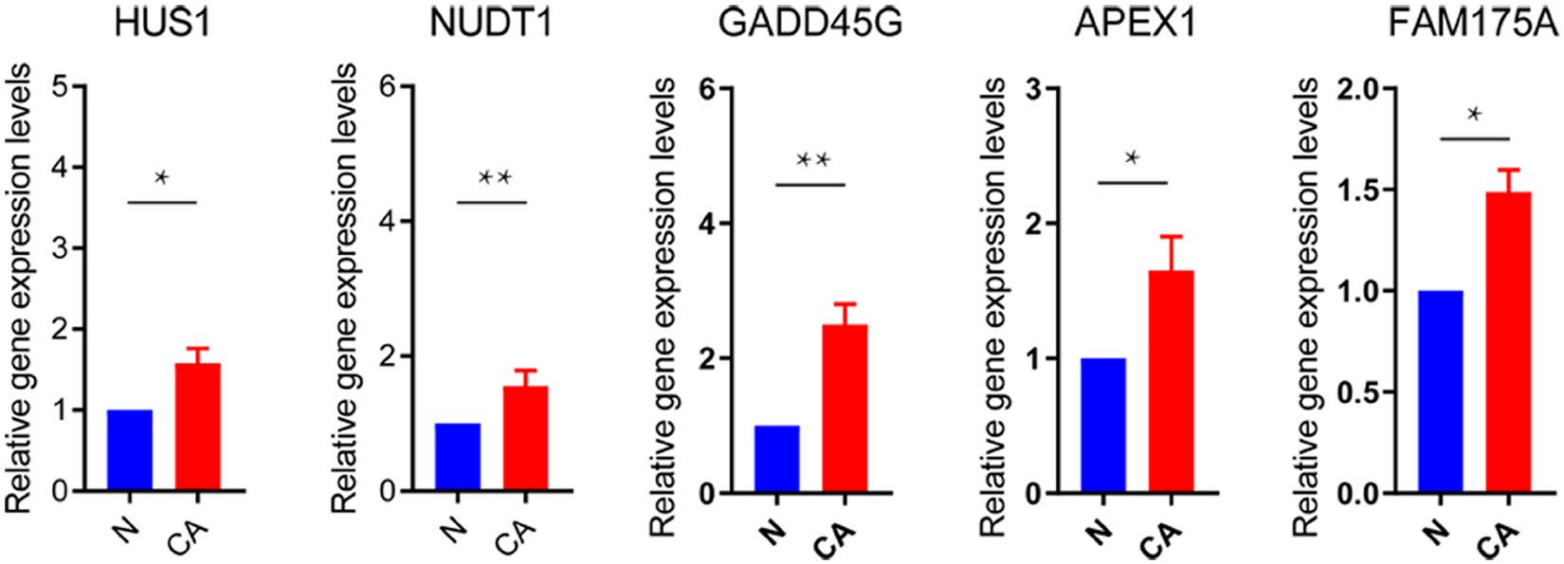
**RT-qPCR validation of DDR-related signature genes**. *P < 0.05; **P < 0.01. N: normal controls; CA: cancer.

**Figure 3.**
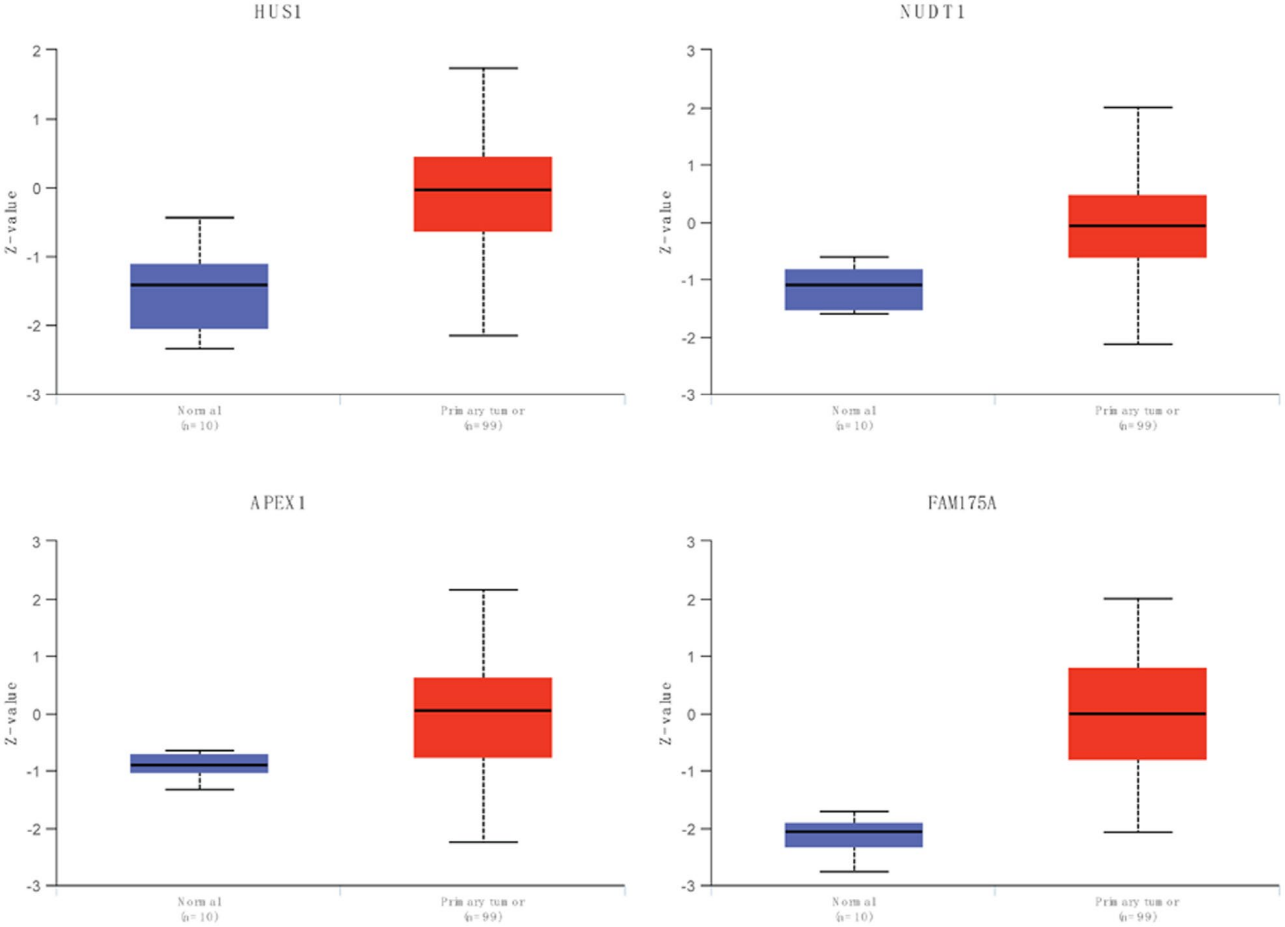
**Protein expression of DDR-related signature genes**. ***P < 0.001.

To explore the role of DDR-related genes in predicting the survival of glioma patients, 23 prognosis-related genes were identified from univariate Cox analysis under the screening criteria of P value < 0.05 (Figure 1(A)). The expression profiles of the above 23 prognostic-related DDR genes were analyzed by LASSO regression analysis. According to the optimal value of λ, 10 DDR-related signature genes were identified, including HUS1 checkpoint clamp component (HUS1), EP300 interacting inhibitor of differentiation 3 (EID3), methylguanine methyltransferase (MGMT), tyrosine 3-monooxygenase/tryptophan 5-monooxygenase activation protein gamma (YWHAG), nudix hydrolase 1 (NUDT1), growth arrest and DNA damage-inducible gamma (GADD45G), apurinic/apyrimidinic endodeoxyribonuclease 1 (APEX1), PMS1 homolog 1, mismatch repair system component (PMS1), SNF2 histone linker PHD RING helicase (SHPRH), and family with sequence similarity 175 member A (FAM175A) (Figure 1(B–D)). Kaplan-Meier survival analysis was performed on signature genes alone. It was found that four genes, EID3, MGMT, NUDT1 and PMS1, were significantly associated with the prognosis and survival of glioma patients (Figure 1(E–H)). Moreover, the gene expression level analysis by RT-qPCR suggested that among 10 DDR-related signature genes, HUS1, NUDT1, GADD45G, APEX1 and FAM175A were highly expressed in patients with glioma (Figure 2). Moreover, the protein expression of HUS1, NUDT1, APEX1 and FAM175A was up-regulated in the UALCAN database (http://ualcan.path.uab.edu/) (involving 99 cases and 10 normal controls) (Figure 3). Significantly, we built the risk score model based on the formula: risk score = −0.04* PMS1 − 0.247*FAM175A − 0.071*SHPRH + 0.019*NUDT1 + 0.157*HUS1 + 0.091*YWHAG − 0.005*GADD45G + 0.112*MGMT + 0.119*EID3 − 0.036*APEX1.

### 3.2. Validity of risk score model

**Figure 4.**
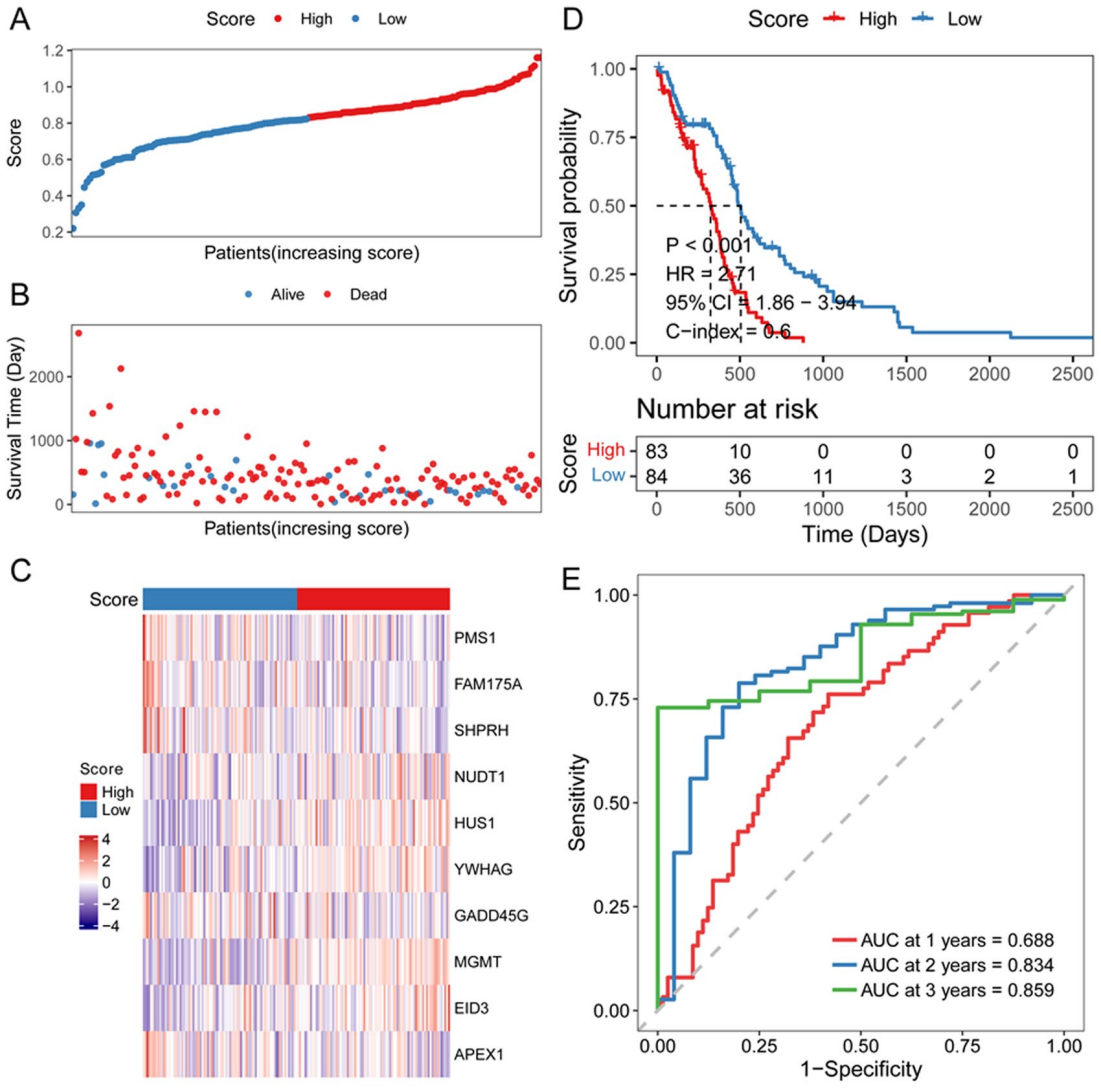
**Efficiency assessment of risk score model**. A: distribution of the risk score for each sample in the TCGA cohort. B: survival of each patient. C, expression heat map of DDR-related signature genes in different risk sore groups. D: Kaplan-Meier survival analysis in each sample. E: time-dependent ROC analysis.

To access the efficiency of the risk score model, glioma patients were divided into two groups according to the median risk score (Figure 4(A)). The relationship between each patient’s survival and risk score was shown using a scatter plot (Figure 4(B)). Thus it can be seen that with a higher risk score, the patient survival was shorter. The heat map of signature genes expression level showed that NUDT1, HUS1, YWHAG and MGMT were highly expressed in high-risk score groups, and PMS1, FAM175A and GADD45G were lowly expressed (Figure 4(C)). High-risk score patients had significantly lower OS than low-risk score patients (Figure 4(D)). To assess the predictive efficiency of the prognostic model in 1-, 2-, and 3-year survival, we performed ROC curve analysis. The AUC was 0.688 at 1 year, 0.834 at 2 years, and 0.859 at 3 years (Figure 4(E)).

### 3.3. Stability validation of the risk score model

**Figure 5.**
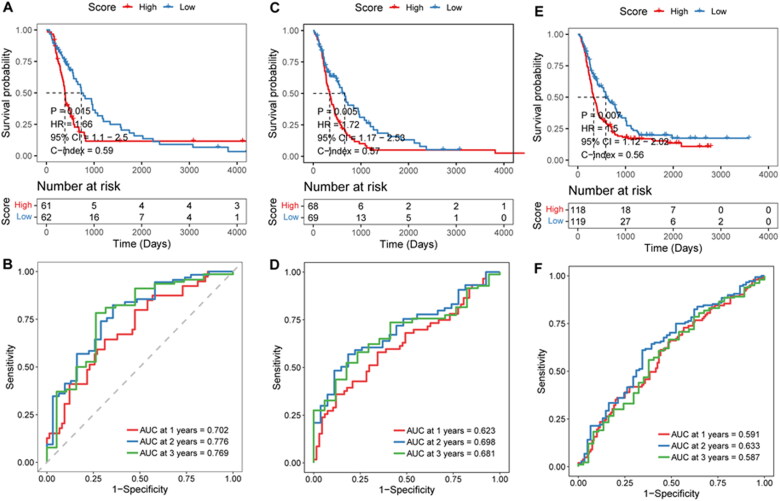
**Effectiveness evaluation of risk score in different cohorts**. A–B, Kaplan-Meier survival and time-dependent ROC analysis in the mRNA-array_301 cohort. C-D, Kaplan-Meier survival and time-dependent ROC analysis in the mRNA-array_325 cohort. E–F, Kaplan-Meier survival and time-dependent ROC analysis in the mRNA-array_693 cohort.

To test the stability of the prognostic risk score model, the mRNA-array_301 cohort, mRNAseq_325 cohort, and mRNAseq_693 cohort were selected to perform the stability analysis. In the mRNA-array_301 cohort, patients in the low-risk score groups had a longer survival time (Figure 5(A)). AUC was 0.688 at 1 year, 0.834 at 2 years, and 0.859 at 3 years (Figure 5(B)). Results of the mRNA-array_301 cohort suggested that high-risk score groups had a shorter survival time (Figure 5(C)). AUC was 0.623 at 1 year, 0.698 at 2 years, and 0.682 at 3 years (Figure 5(D)). Survival analysis in mRNAseq_693 cohort was consistent with the above two cohorts (Figure 5(E)). ROC curve analysis of mRNAseq_693 cohort indicated that AUC was 0.591 at 1 year, 0.633 at 2 years, and 0.587 at 3 years (Figure 5(F)).

### 3.4. Independent prognostic value of risk score

**Figure 6.**
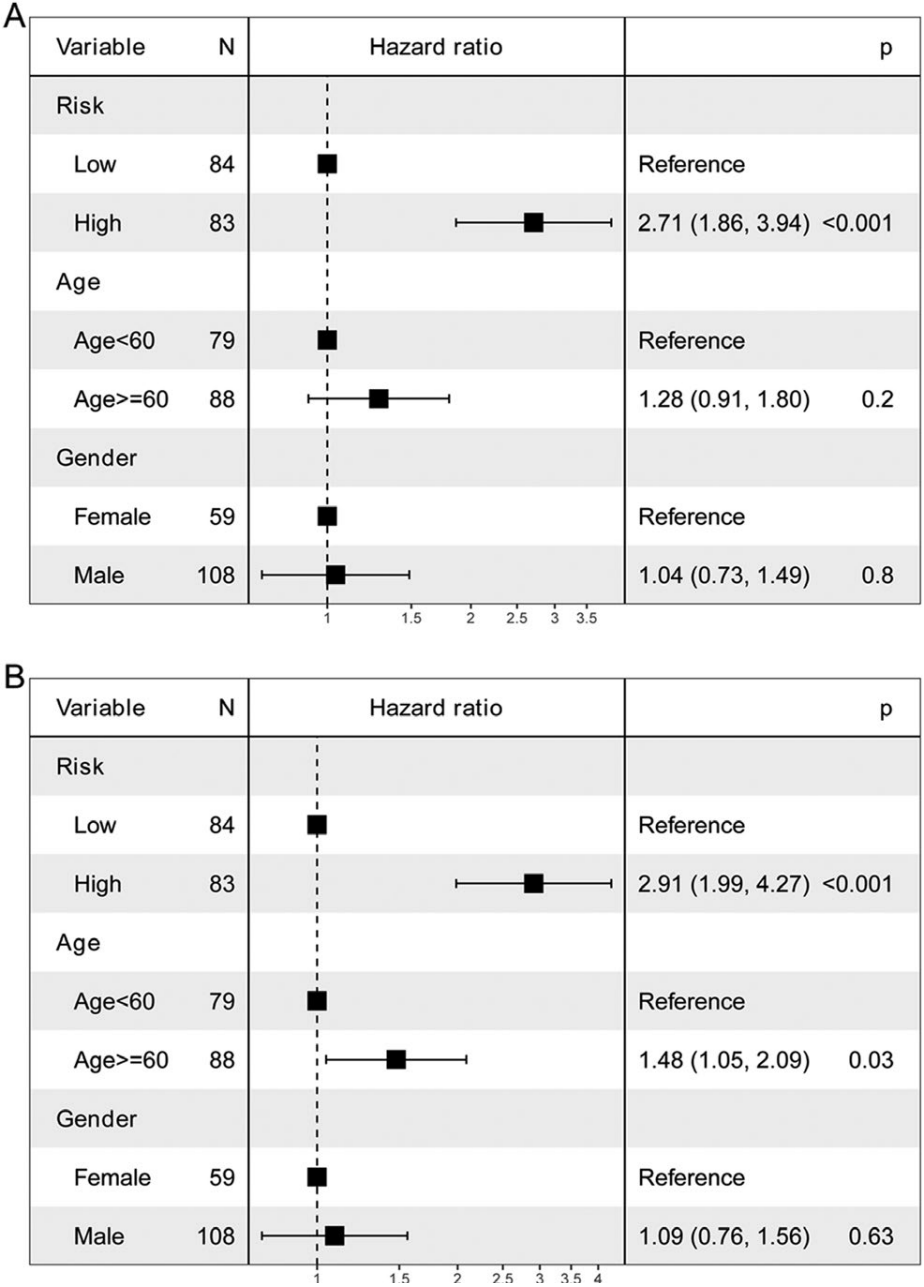
**Independent prognostic value of the risk score**. A: univariate Cox analysis. B: multivariate Cox analysis.

Univariate and multivariate Cox analyses of variables were employed to determine whether the risk score was an independent prognostic factor for OS. Risk score was significantly associated with OS in univariate Cox analysis (Figure 6(A)). After adjustment for other confounders, risk score remained an independent predictor of OS (Figure 6(B)).

### 3.5. Applicability of risk score model in different clinical subgroups

**Figure 7.**
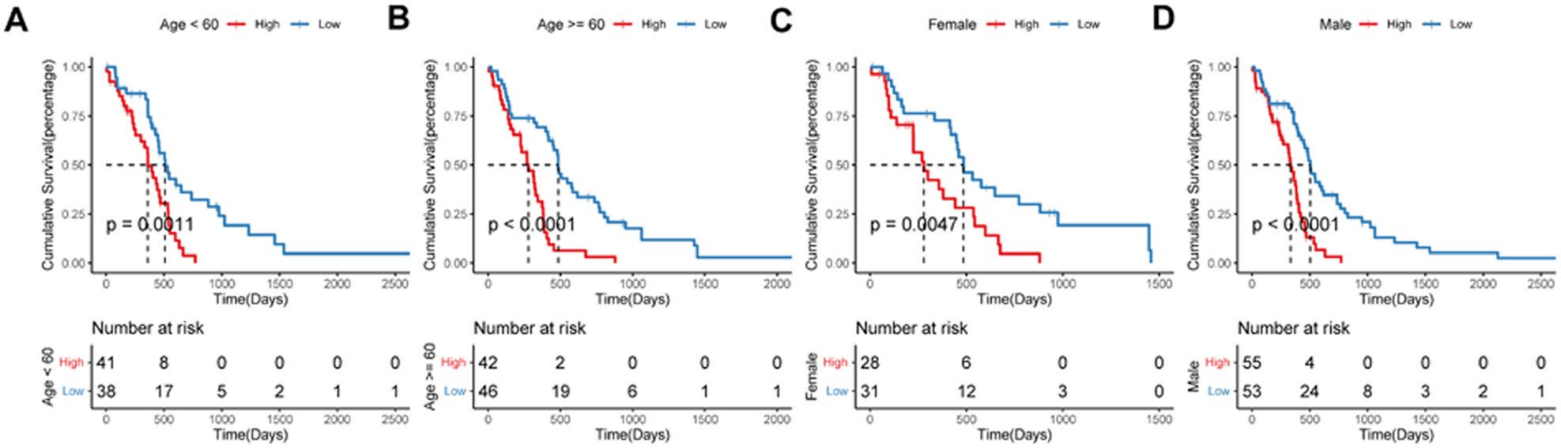
**Prognostic efficacy of the risk score model in different clinical subgroups**. A–B, Kaplan-Meier survival analysis in age subgroup. C–D, Kaplan-Meier survival analysis in sex subgroup.

To explore the applicability of the constructed prognostic model in different glioma populations, subgroup analyses were performed. Patients in the high-risk score group had a worse prognosis than those in the low-risk score group in all subgroups (Figure 7(A–D)).

### 3.6. Relationship between risk score and TMB

**Figure 8.**
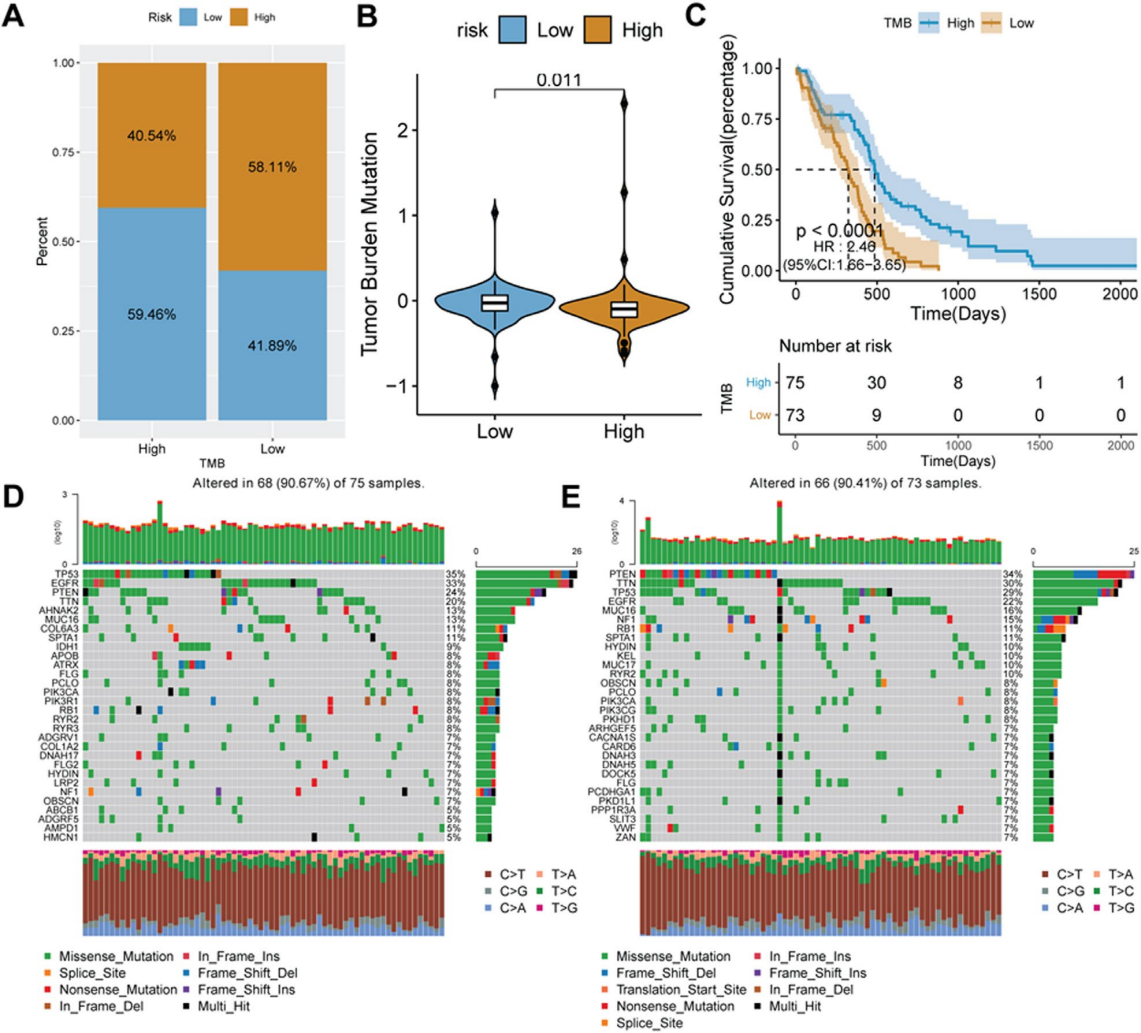
**Relationship analysis of risk score and TMB**. A, the distribution ratio of the risk score in different TMB score groups. B, distribution of TMB score in different risk score groups. C, Kaplan-Meier survival analysis in different TMB scores groups. D–E, gene mutation waterfall charts in the low-risk score and high-risk score groups.

To explore the intrinsic link between TMB and risk score, we calculated the TMB score. Tumor samples were divided into high- and low-TMB score groups. Interestingly, we found that the proportion of glioma patients with low-risk scores was higher in the high-TMB score group, reaching 59.46% (Figure 8(A)). Mean TMB score in the low-risk score group was significantly higher than that in the high-risk score group (*p* = 0.011) (Figure 8(B)). Patients with high-TMB scores had longer survival (Figure 8(C)). To further evaluate the distribution of somatic variants in glioma driver genes between different risk score groups, the top 30 driver genes with the highest frequency of changes were compared. The results showed that in the high-risk score group, TP53 had the highest mutation rate, while in the low-risk score was PTEN (Figure 8(D, E)).

### 3.7. Relationship analysis of risk score and immune cell infiltration

**Figure 9.**
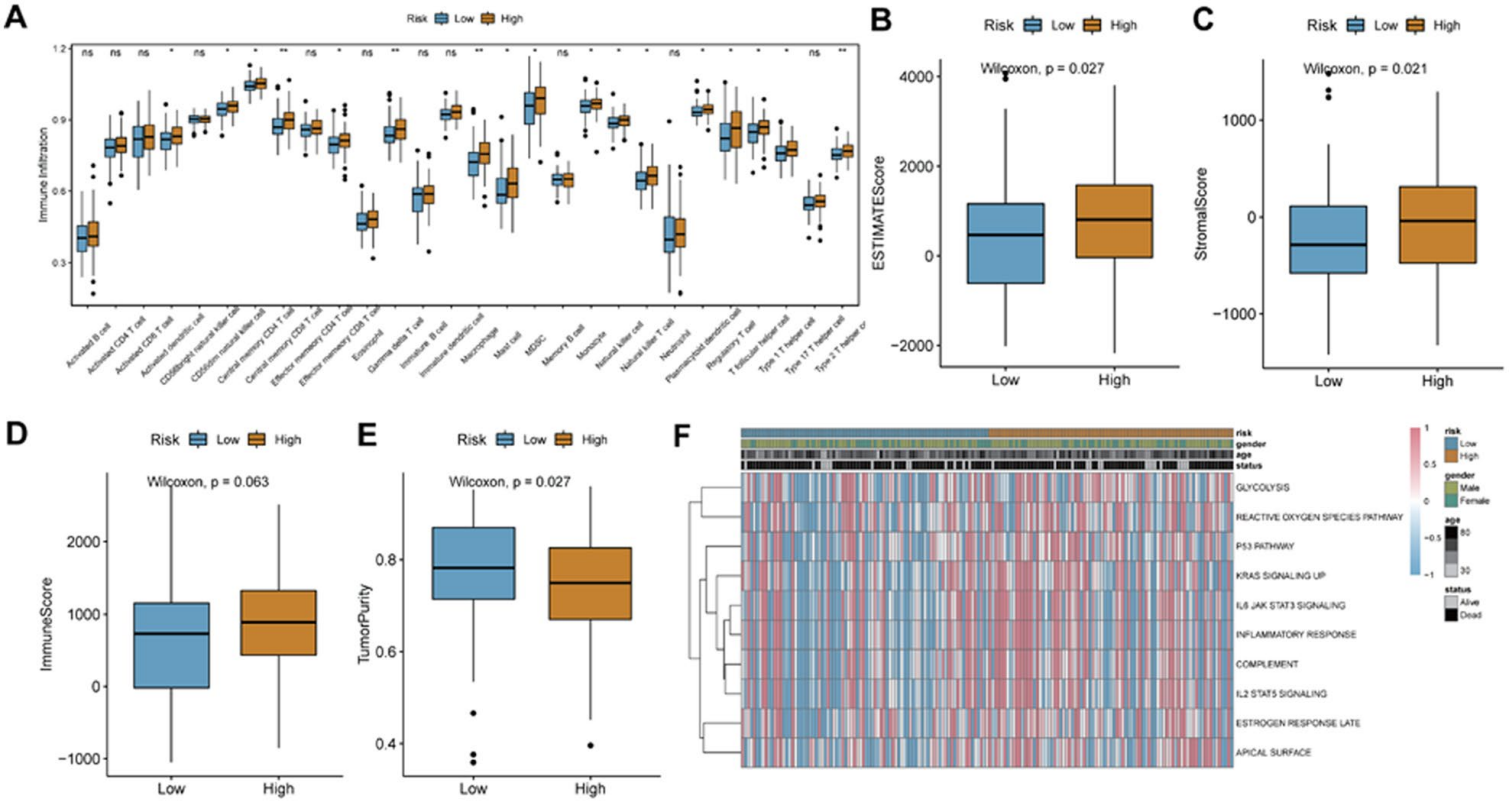
**Relationship analysis of risk score and immune cell infiltration**. A, box plot of immune cell infiltration between high-and low-risk score groups; B: difference in immune score between high-and low-risk score groups. C, difference in ESTIMATE score between high-and low-risk score groups. D, difference in stromal score between high-and low-risk score groups. E, difference in tumor purity between high-and low-risk score groups. F, GSVA analysis of differentially expressed pathways. *P < 0.05; **P < 0.01; ns: not significant.

To analyze the relationship of the risk score and the TIME, we evaluated the status of 23 immune cell infiltrations in the TCGA_ glioma tumor sample dataset. Infiltration degree of most immune cells in the high-risk score group was significantly higher than that in the low-risk score group, such as activated dendritic cells, central Memory CD8+ T cells, central memory CD4+ T cells, etc. (Figure 9(A)). Immune score, stromal score, tumor purity and ESTIMATE score were calculated for each glioma patient. In the high-risk score group, the ESTIMATE score and stromal score were significantly higher than those of the low-risk score group (Figure 9(B, C)). Immune score of the high-risk score group was higher than that of the low-risk score group, but it was not significant (Figure 9(D)). While the tumor purity of the high-risk score group was significantly higher compared to low-risk score group (Figure 9(E)). Moreover, results of GSVA analysis showed that only 10 signaling pathways were identified under the screening criteria of P value < 0.05. Moreover, IL6 JAK STAT3 and IL2 STAT5 signaling pathways were more active in the high-risk score group (Figure 9(F)).

### 3.8. Relationship analysis of risk score and immunotherapy

**Figure 10.**
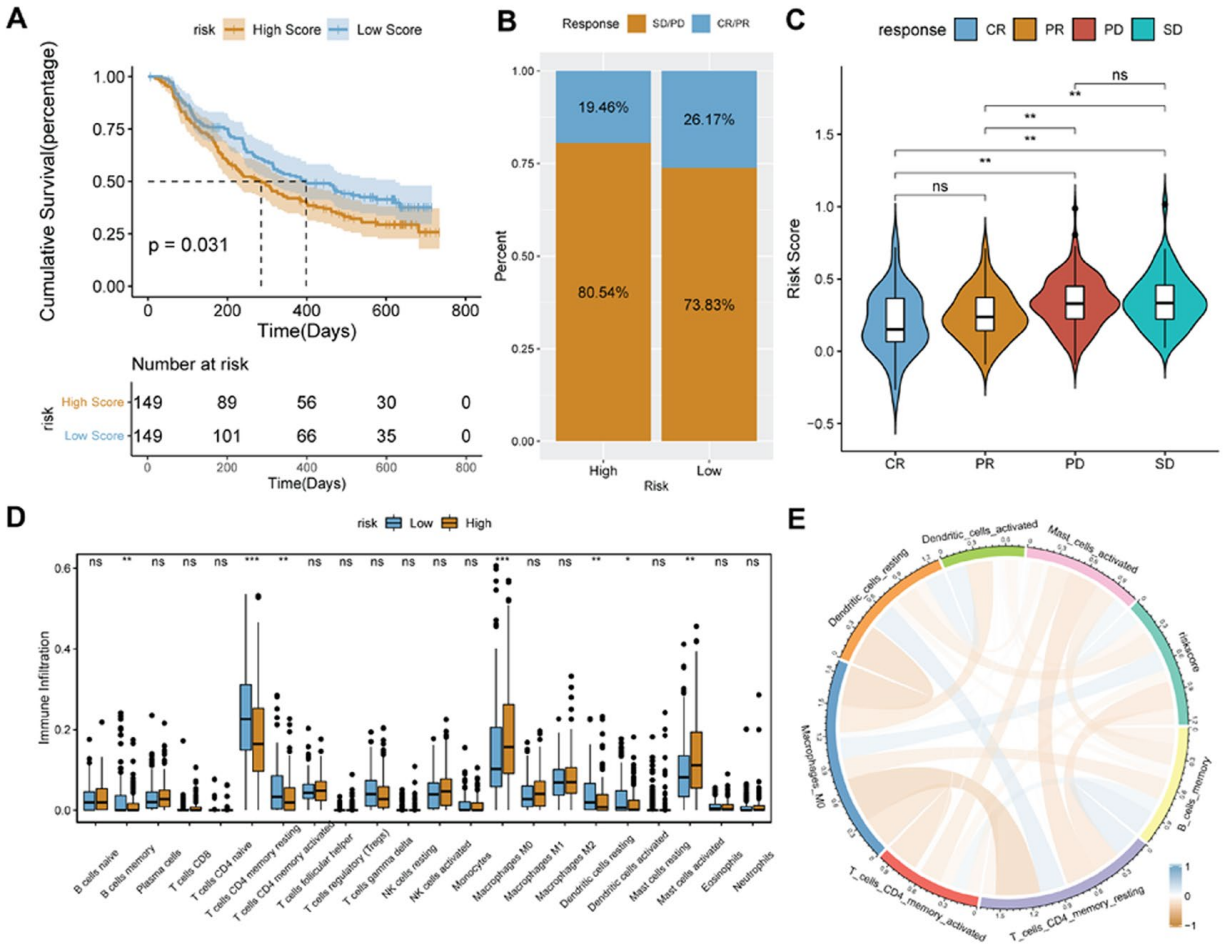
**Prediction of immunotherapy response in the IMvigor 210 cohort**. A, Kaplan-Meier survival analysis between high- and low-risk score groups. B, histogram of immunotherapy response ratios between high- and low-risk score groups in the IMvigor210 cohort. C, differences in risk scores according to the efficacy of immunotherapy. D, immune cell infiltration between high- and low-risk score groups. E, correlation between risk score and immune cells. **p < 0.01; ns: not significant.

Identification of new predictive markers is critical for effective immunotherapy. We used the IMvigor210 cohort, which uses anti-PD-L1 immunotherapy, to explore whether DDR-related signature genes can predict the benefit of immunotherapy. Patients with high-risk score had a worse prognosis than patients with low-risk score (Figure 10(A)). The proportion of responders to immunotherapy (CR/PR; complete remission, CR; partial remission, PR) was significantly higher in the low-risk score group (Figure 10(B)). Risk score was significantly higher in patients with stable disease (SD) or progressive disease (PD) than in patients with CR or PR (Figure 10(C)). Infiltration degree of B cells memory cells, CD4+ Memory T cell and dendritic cells in the high-risk score were significantly lower, while the enrichment degree of Macrophages M0 cells in the low-risk score group was significantly lower (Figure 10(D)). In addition, risk score was positively related to the immune cell infiltration score (Figure 10(E)).

### 3.9. Relationship analysis of risk score and sensitivity of chemotherapy

**Figure 11.**
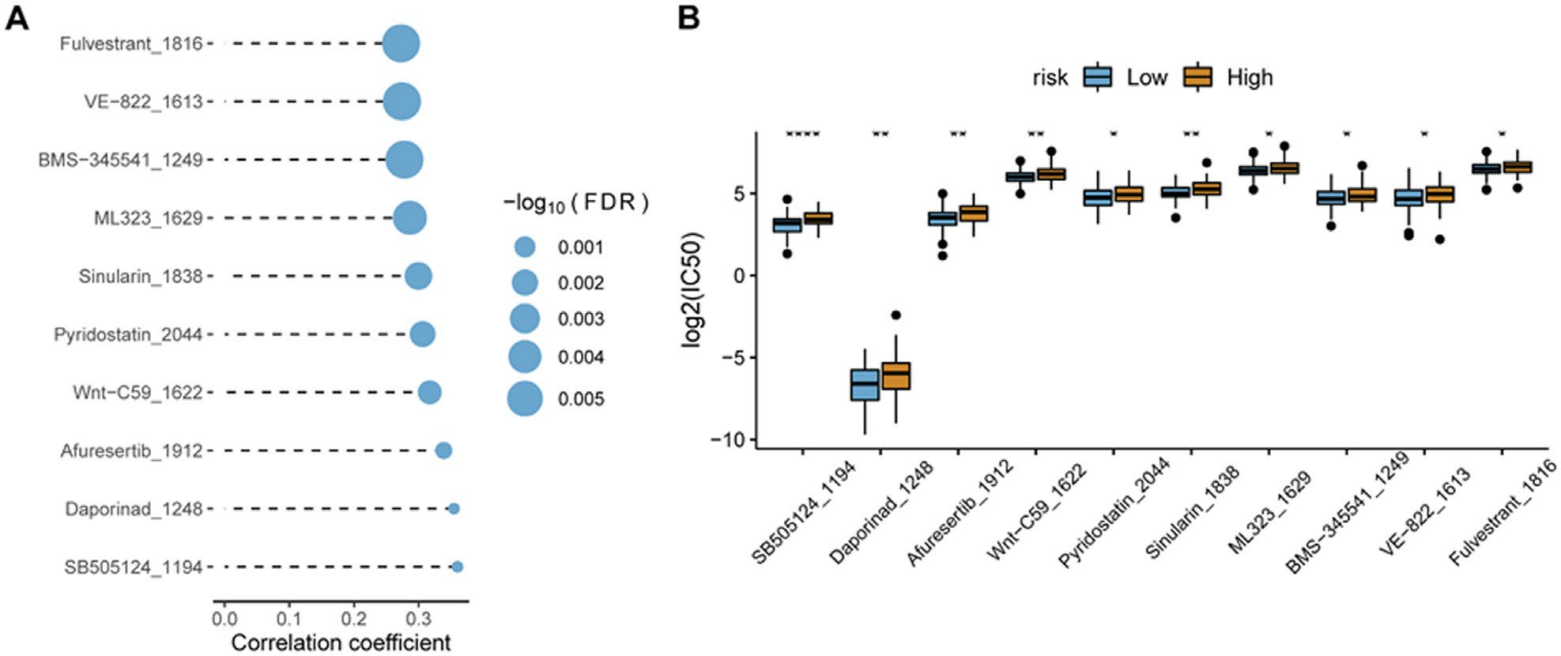
**Relationship analysis of risk score and drug sensitivity**. A, spearman correlation between drug IC50 and risk score. B, difference in drug IC50 values between high- and low-risk score groups. *p < 0.05; **p < 0.01; ^****^p < 0.0001.

In the GDSC database, many clinically functional genes are targets of anticancer drugs. To further assess the effects of the risk score on drug response, the spearman correlation analysis of the IC50 value of the drug and the risk score was performed. The IC50 value of the drug was significantly positively related to the risk score (Figure 11(A)). Interestingly, the IC50 value of the drug was also significantly lower in the low-risk score group than in the high-risk score group (Figure 11(B)).

### 3.10. Knockdown of NUDT1 reduces tumor cell migration and invasion

**Figure 12.**
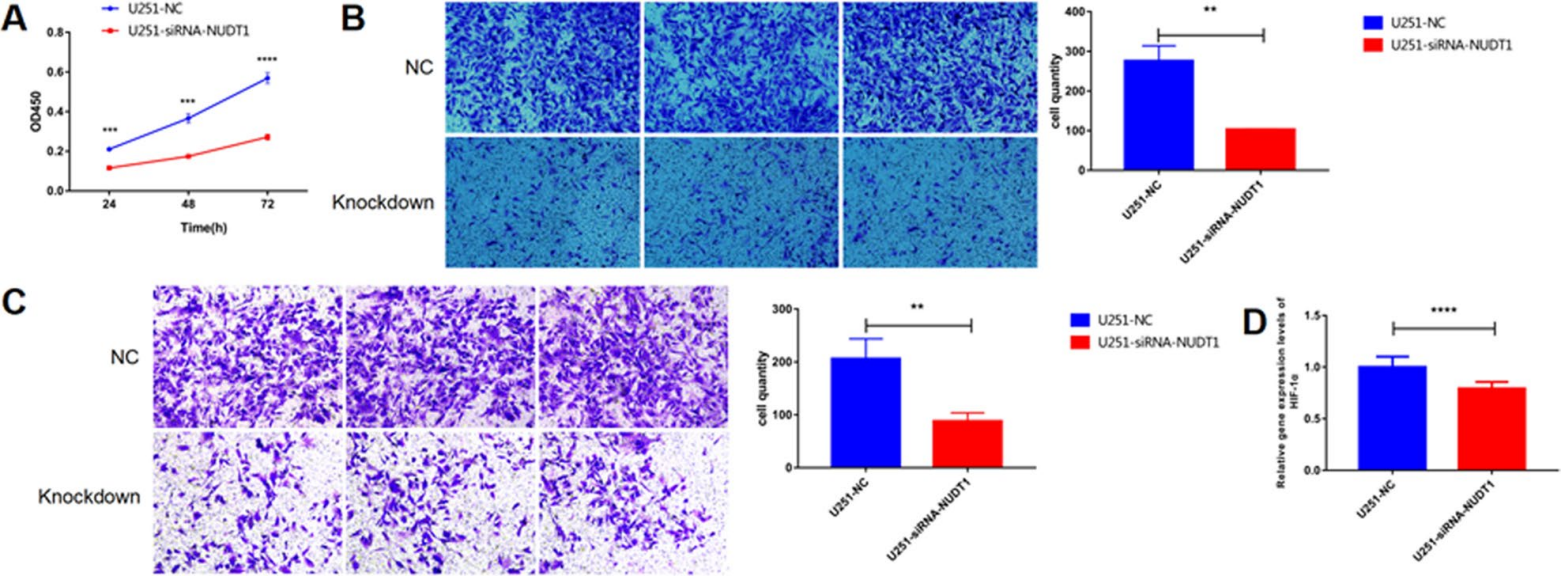
**The effect of NUDT1 on cell proliferation, migration and invasion**. In view of analysis results and related literature reports, one of DDR related gene, NUDT1, was used for further molecular mechanism analysis. From the CCK-8 experiment, we found that NUDT1 promoted tumor cell proliferation. A, the effect of NUDT1 on cell proliferation. B, the effect of NUDT1 on cell migration. C, the effect of NUDT1 on cell invasion. D, the effect of NUDT1 on mRNA expression of HIF-1α. **p < 0.01; ***p < 0.001; ^****^p < 0.0001.

In view of analysis results and related literature reports, one of DDR related gene, NUDT1, was used for further molecular mechanism analysis. From the CCK-8 experiment, we found that NUDT1 promoted tumor cell proliferation at 24, 48 and 72h after transfection (Figure 12(A)). Cell migration analysis (Figure 12(B)) showed that knockdown of NUDT1 significantly reduced the migration of tumor cells after transfection. The result of Transwell assay (Figure 12(C)) also showed that knockdown of NUDT1 reduced the migration and invasion of tumor cells. It is noted that the knockdown of NUDT1 significantly reduced the mRNA expression of hypoxia-inducible factor 1 subunit alpha (HIF-1α) (Figure 12(D)).

## 4. Discussion

Recent developments in the molecular mechanisms of the cell cycle have led to renewing interest in gaining a deeper understanding of glioma [23]. Furthermore, interpreting cancer as a genetic disease has been replaced by a disorder of cell cycle regulation [24,25]. Glioma is a malignant tumor [26]. At present, its pathogenesis is not clear. Genetics, diet, radiation, infection with cytomegalovirus, and genetic polymorphisms of DNA repair-related genes are all associated with the development of glioma [27–29]. Accumulating evidence suggests that DDR-related genes play key roles in glioma [3]. Excitingly, glioma treatment strategies are currently focused on generating overwhelming DNA damage and inhibiting repair mechanisms. In this study, we analyzed DDR-related genes using a range of bioinformatic analysis methods. In addition, multivariate Cox and LASSO regression algorithms were used to construct a risk score model based on DDR to identify novel biomarkers in glioma patients. The model can well predict the prognosis of glioma patients, which is of great significance in the diagnosis and treatment of patients in clinical practice.

In this study, we identified 10 DDR prognostic signature genes and constructed a risk score model. Among these, EID3, MGMT, PMS1, and NUDT1 were significantly related to the prognosis and survival of glioma patients, and the high-risk score group had a significantly shorter survival time. EID3, belonging to a member of the IED family, is involved in regulating tumor development in different cancer types [30,31]. EID3 showed a trend of low expression in the brain tissue of glioma patients [32]. Interestingly, high expression of EID3 in glioma is associated with poorer prognosis [32]. MGMT encodes a protein that repairs DNA [33]. Significantly, MGMT is associated with a variety of tumors, such as glioma and glioblastoma [34,35], esophageal adenocarcinoma [36], liver cancer [37], and thyroid cancer [38], etc. In glioma, low DDR scores are associated with MGMT methylated status [10]. In addition, TMZ resistance in glioblastoma multiforme is mediated by MGMT. The report suggested that the methylation of MGMT was able to generate a good response to TMZ treatment [39]. PMS1 is related to tumor susceptibility [40]. NUDT1 can hydrolyze oxidized purines and prevent them from being added to DNA strands during DNA repair [41]. Role of NUDT in tumorigenesis is so far ambiguous. Originally thought to function as a sanitizing enzyme of the oxidized nucleotide pool and thus important for cancer cell survival [42,43]. NUDT1 plays an important role in eliminating oxidized nucleotides [44].

The above literature has fully demonstrated that the regulation of DDR-related genes on glioma progression. We adopted a comprehensive analysis approach to fully validate the validity of the risk score model for prognosis prediction in different cohorts. *In vitro* experiments, we further investigated the molecular mechanism of NUDT1. The result showed that knockdown of NUDT1 reduced tumorigenesis. Furthermore, knockdown of NUDT1 significantly decreased the expression levels of HIF-1α. In solid tumours, hypoxia is common and may alter the response of tumour cells to NUDT1 inhibitors, which have an impact on redox signaling [45]. It is suggested that NUDT1 may play an important role in tumorigenesis of glioma by activating the expression of HIF-1α.

TMB analysis is important for an in-depth understanding of the pathogenesis of tumors. On the one hand, TMB driver gene mutations can result in tumorigenesis. On the other hand, a large number of somatic mutations can generate neoantigens, which can activate CD8+ cytotoxic T cells, so as to exert T cell-mediated anti-tumor effect [46]. In this work, patients in high-TMB score groups had a better prognosis. During tumorigenesis, malignant cells diversify become more heterogeneous [47]. Therefore, an accurate understanding of tumor immune heterogeneity is important to effective treatments [21]. Interestingly, the immune infiltration analysis indicated that reduced immune cell infiltration in the tumor microenvironment in high-risk groups may contribute to poorer patient outcomes.

To further evaluate the validity of the risk score model, we performed immunotherapy analysis and chemotherapeutic drug sensitivity analysis in different risk score groups. Excitingly, the low-risk score group was more sensitive both in response to immunotherapy and chemotherapy, which may be one of the reasons for the better prognosis. However, this work also has limitations. This paper only considers the impact of DDR-related genes on glioma from the transcriptional level, and some other factors, such as the environment, diet, genetics, gene modification, etc., are not considered.

## 5. Conclusion

DDR-related risk score model built-in this work has good predictive performance for glioma.

## Supplementary Material

Supplemental Material
