# Supplementary material for: Uncover DNA damage and repair-related gene signature and risk score model for glioma
Source: Ann Med. 2023 Apr 22;55(1):2200033. doi: 10.1080/07853890.2023.2200033 (PMC10124985; doi:10.1080/07853890.2023.2200033)
Supplement: Supplemental Material [file IANN_A_2200033_SM9070.docx]

**Supplementary Table 1 Gene primers used in the RT-qPCR**

| **Gene** | **Sequence (5'to3')** | **Production (bp)** |
| --- | --- | --- |
| ACTB-F | CATGTACGTTGCTATCCAGGC | 250 |
| ACTB-R | CTCCTTAATGTCACGCACGAT |  |
| HUS1-F | GAACGGAAGGAAGCAGTGAG | 153 |
| HUS1-R | TGTTCCAGCTCACACCACAT |  |
| NUDT1-F | GCTCATGGACGTGCATGTCTT | 140 |
| NUDT1-R | GTGGAAACCAGTAGCTGTCGT |  |
| APEX1-F | CAATACTGGTCAGCTCCTTCG | 88 |
| APEX1-R | TGCCGTAAGAAACTTTGAGTGG |  |
| GADD45G-F | GACCTGCAVTGCATCCTCAT | 170 |
| GADD45G-R | ACCACGTCGATCAGACCAAG |  |
| FAM175A-F | TCAGGCGAAGTAAATGAGCAAG | 80 |
| FAM175A-R | CGGAATTTGTACCAACCTACCAC |  |
